# Factors Influencing an Early Career Choice of Child and Adolescent Psychiatry Among Chinese Medical Students: An Exploratory Survey

**DOI:** 10.1007/s40596-025-02207-6

**Published:** 2025-09-05

**Authors:** Meirong Pan, Xinxin Yue, Ni Tang, Qingjiu Cao, Tianmei Si

**Affiliations:** Peking University Sixth Hospital, Peking University Institute of Mental Health, NHC Key Laboratory of Mental Health (Peking University), National Clinical Research Center for Mental Disorders (Peking University Sixth Hospital), Haidian District, No. 51 Hua Yuan Bei Road, Beijing, 100191 China

**Keywords:** Child and adolescent psychiatry, Recruitment, Residency training, Fellowship

## Abstract

**Objective:**

The study aimed to explore factors influencing Chinese medical students’ career choices, especially in choosing child and adolescent psychiatry (CAP) as their specialty.

**Method:**

A 23-item online survey was conducted among third-year medical students at Peking University Health Science Center to explore the factors currently influencing their career choices, as well as their preferences and factors that influence the choice of CAP as a career specialty. Their preferences for CAP-focused curricula were also surveyed.

**Results:**

Of the 169 students surveyed, 149 (88.17%) responded. Over 80% of the students began contemplating their future career choices, even before their undergraduate studies commenced. Sixty (40.27%) students showed moderate to high interest in CAP, and the top three factors that rendered CAP most attractive included interest in helping society (58.33%), having opportunities to work with children and adolescents (56.67%), and working in an interesting field (53.33%). The primary obstacle to choosing CAP as a specialty was the burden of working with patients and their parents (70.79%). Among the students interested in CAP, 85.00% were interested in engaging with CAP through clinical internships and practical training. Curricula related to psychotherapy, consultation liaison, and psychopharmacology were identified as the most attractive areas.

**Conclusions:**

Positive attitudes toward CAP were found among the responders. To better align with the career development needs of medical students and promote workforce development in the CAP specialty, early career exposure to CAP, such as encompassing clinical clerkships, specialized internships, and interdisciplinary education, should be given more consideration in undergraduate curriculum systems.

Child and adolescent mental health (CAMH) is a global priority [1], with a high prevalence of mental disorders and significant associated disability. The worldwide-pooled prevalence is 13.4% [2], accounting for 20.27% of disability-adjusted life years (DALYs) from all causes [3]. In China, the prevalence among school-aged children and adolescents was estimated at 17.5% in 2015 [4], and during the COVID-19 pandemic, the rates of anxiety and depressive symptoms increased to 36.7% and 57.0%, respectively [5]. With at least 50 million children and adolescents requiring professional help, access to effective treatment and support is critically important [6]. However, access to mental health treatment for this age group remains inadequate globally, since the estimated treatment rate was only 38%, highlighting the vast gap between CAMH needs and the availability of CAMH resources [7]. In China, fewer than 10% of depressed children are treated [8], identifying crucial information for resource-limited settings.

Many factors contribute to the lack of CAMH services access, including structural barriers (e.g., workforce shortages, geographic disparities), sociocultural stigma, economic constraints, and systemic inequities disproportionately affecting marginalized populations [9, 10]. Among these challenges, the child and adolescent psychiatry (CAP) workforce shortage is particularly critical. The American Academy of Child and Adolescent Psychiatry (AACAP) estimates that a country needs 47 child and adolescent psychiatrists per 100,000 youth aged 0 to 19, whereas the current number is relatively small and varies in different regions: up to 9.75 in the USA, 5.5 in other high-income countries, and less than 0.1 in middle- and low-income countries [10]. In China, there were fewer than 500 full-time child and adolescent psychiatrists in 2019 [11]. The workforce shortage of mental health professionals may be associated with an increased rate of youth suicide [12]. Thus, strategically expanding the CAP workforce globally, particularly in middle- and low-income countries, is paramount to bridging the gap between demand and supply, ensuring equitable access to CAMH services worldwide.

In China, medical education programs are designed to provide training for future physicians [13]. Currently, most medical students complete 5 years of undergraduate medical education to earn a bachelor’s degree, followed by a 3-year psychiatry residency training to become psychiatrists. After completing residency, they may choose to pursue subspecialty training. During the undergraduate phase, psychiatry is typically taught in the third or fourth year of medical school and consists of theoretical courses, problem-based learning, case-based learning, clerkships, and internships. However, few CAP-focused courses have been tailored for medical students, and the broader curriculum does not emphasize CAP as a distinct specialty. This lack of targeted exposure may contribute to limited interest in CAP among Chinese medical students.

Globally, addressing the shortage of CAP professionals has necessitated interventions to increase early exposure to CAP within medical education. Such exposure can make CAP more appealing to medical students as they deliberate over various specialties, explore avenues, seek opportunities, and ask for mentorship for their future endeavors [14]. The third year of medical school is particularly pivotal for career decision-making, as students begin clinical rotations that significantly shape their professional interests and choices [15]. Recent studies have shown that during medical courses, increased exposure to CAP may increase the number of medical students choosing CAP as a career specialty, such as through mentorship networks, clinical experiences, and research opportunities [16–18]. Students attending a summer immersion in CAP [19] or a case-based seminar during the third year during clerkships [20] also reported being more likely to pursue a career in CAP. In addition, in the USA, multiple CAP-related courses are offered as elective courses, such as Child and Adolescent Psychopathology or Child Development, designed to heighten students’ awareness of and interest in CAP [21].

Despite these global insights, research specifically focused on Chinese medical students’ attitudes toward CAP remains limited. To date, precise data on medical graduates’ subspecialty choices are still lacking, which hinders a comprehensive understanding of CAP’s appeal as a career path. Extrapolating from international benchmarks and China’s clinician density (0.05 CAP specialists per 100,000 children), we estimate that < 0.5% of psychiatric trainees enter CAP, a rate reflecting systemic underinvestment in training and incentives. Preliminary studies delved into students’ attitudes toward psychiatry, consistently yielding negative outcomes primarily attributed to dissatisfaction with financial compensation and perceived prestige [22]. Zhang et al. explored medical students’ career preferences and their underlying determinants in psychiatry and revealed that personal interest and undergoing a psychiatry clerkship positively correlate with students’ choice of psychiatry as a career [23]. However, no studies have delved into the attitudes and preferences of Chinese medical students toward CAP as a distinct specialty, nor have they identified the pivotal factors that shape their career choices in this field. This gap underscores the need for targeted research and educational interventions to foster interest in CAP among Chinese medical students.

To fill this gap, this study aimed to explore the current career choices of Chinese medical students and the factors that influence these choices, the attractiveness and obstacles of choosing CAP as a specialty, and students’ preference for CAP-focused courses during their undergraduate education. We sought to gather empirical evidence that could strengthen the implementation of CAP-focused educational initiatives and attract more medical students to select CAP as their career choice, which could help expand CAMH resources.

## Methods

### Participants and Procedure

An online survey was administered to third-year medical students at Peking University Health Science Center. The survey was delivered via an electronic questionnaire, and the students could complete the questionnaire on their mobile phones by scanning a QR code. The questionnaire took approximately 10 to 15 min to complete. The data were collected anonymously and were posted twice in the class-specific announcement group.

The participants were well informed of this survey by the investigators and signed informed consent forms before they had access to the electronic questionnaire. This study received ethical approval from the Medical Ethics Committee of Peking University Sixth Hospital, with the approval number ((2024) Ethics Review No. (68)). All procedures followed the ethical principles of the Declaration of Helsinki and relevant regulations for human subject research.

### Questionnaire Design and Contents

The questionnaire was designed based on a comprehensive review of existing surveys related to medical students’ career choices, particularly in CAP [16, 24, 25]. To ensure the relevance and clarity of the questions, the survey was reviewed by a panel of experts, including psychiatrists, medical educators, and researchers with expertise in CAP and medical student training. Their feedback was incorporated to refine the wording and structure of the questionnaire. Additionally, the survey was piloted with a small group of medical students (*n* = 10) under anonymized and confidential conditions to assess comprehension, identify potential ambiguities, and evaluate the time required for completion. Based on the pilot feedback, minor adjustments were made to improve clarity and flow.

The final questionnaire consisted of 23 items covering the following areas: First, we collected demographic information, including age, gender, ethnicity, family annual income, and economic burden. A multiple-choice questionnaire with a scoring system was subsequently used to gather data on students’ current career considerations and their influencing factors, their preferences toward CAP as a potential specialty, the perceived attractiveness and obstacles when choosing CAP as a career specialty, and their preferences for CAP-focused curricula during undergraduate courses.

Students were requested to rate factors influencing their specialty choice on a scale ranging from 0 to 10, where 0 denoted no influence and 10 signified a strong influence. The Likert-scale responses were subsequently treated as continuous variables. For each question, the mean ratings for each question were calculated along with their standard deviation. Additionally, a rating score of 6 or above indicates that the raters presented moderate or high agreement with the selection.

### Statistics

The raw data from survey responses were imported into IBM SPSS Statistics version 26.0 (IBM Inc., Chicago, IL, USA), where they were cleaned and coded for further analysis. Response frequency information was obtained. Descriptive statistics were calculated using means and percentages of responses for demographic variable responses. Descriptive statistics were calculated for the analysis of respondents’ demographics, general interest choices, and relevant appraisals.

## Results

### Demographics Data

Among the 169 third-year medical students surveyed, 149 responded (88.17% response rate). The relevant demographic data are summarized in Table 1. The students were 19–23 years old (21.21 ± 0.61). Among the students, 125 (83.89%) were Han Chinese in ethnicity, 70 (46.98%) were male, and 112 (75.17%) were from families with an annual family income < CNY 300,000. A total of 137 (91.95%) reported “no or low economic burden” (see Table 1).

**Table 1:** Summary of the demographics of the survey respondents

		*N* (%)
Gender (male)		70 (46.98%)
Ethnic group	Han	125 (83.89%)
	Others	24 (16.11%)
Family annual income (CNY)	< 30,000	11 (7.38%)
	30,000–80,000	19 (12.75%)
	80,000–150,000	37 (24.83%)
	150,000–300,000	45 (30.20%)
	300,000–500,000	18 (12.08%)
	500,000–1,000,000	13 (8.72%)
	> 1,000,000	6 (4.03%)
Average monthly consumption (CNY)	< 1000	11 (7.38%)
	1000–2000	55 (36.91%)
	2000–3000	56 (37.58%)
	3000–5000	25 (16.78%)
	> 5,000	2 (1.34%)
Current economic burden	None	86 (57.72%)
	Low level	51 (34.23%)
	Mid-level	9 (6.04%)
	High-level	3 (2.01%)

### Career Choice and Influencing Factors

Among the 149 medical students, 126 (84.56%) had commenced contemplating their future career directions. Notably, 14 (11.11%) began this process before their undergraduate studies commenced, 21 (16.67%) during their first year, and 57 (45.24%) during their second year.

**Fig. 1:**
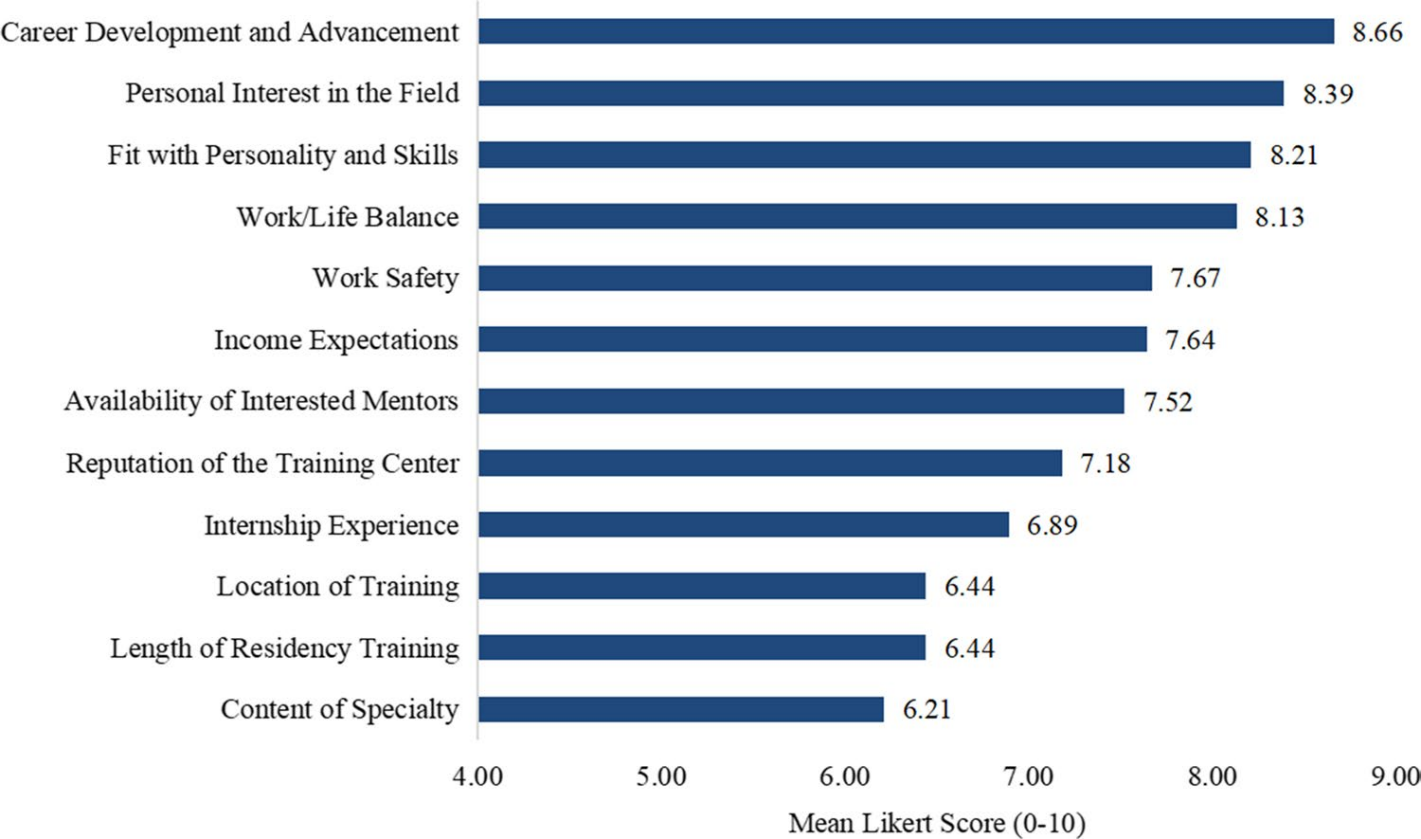
The ranked factors influencing students’ career choices (*N* = 149)

The top three factors influencing students’ subsequent career choice ranked in order of importance were (1) career development and advancement (8.66 ± 1.51); (2) personal interest in the field (8.39 ± 1.72); and (3) fit with personality and skills in the field (8.21 ± 1.84). The detailed ranked scores are listed in Fig. 1.

### Attractiveness and Obstacles to Choosing CAP as a Specialty

**Fig. 2:**
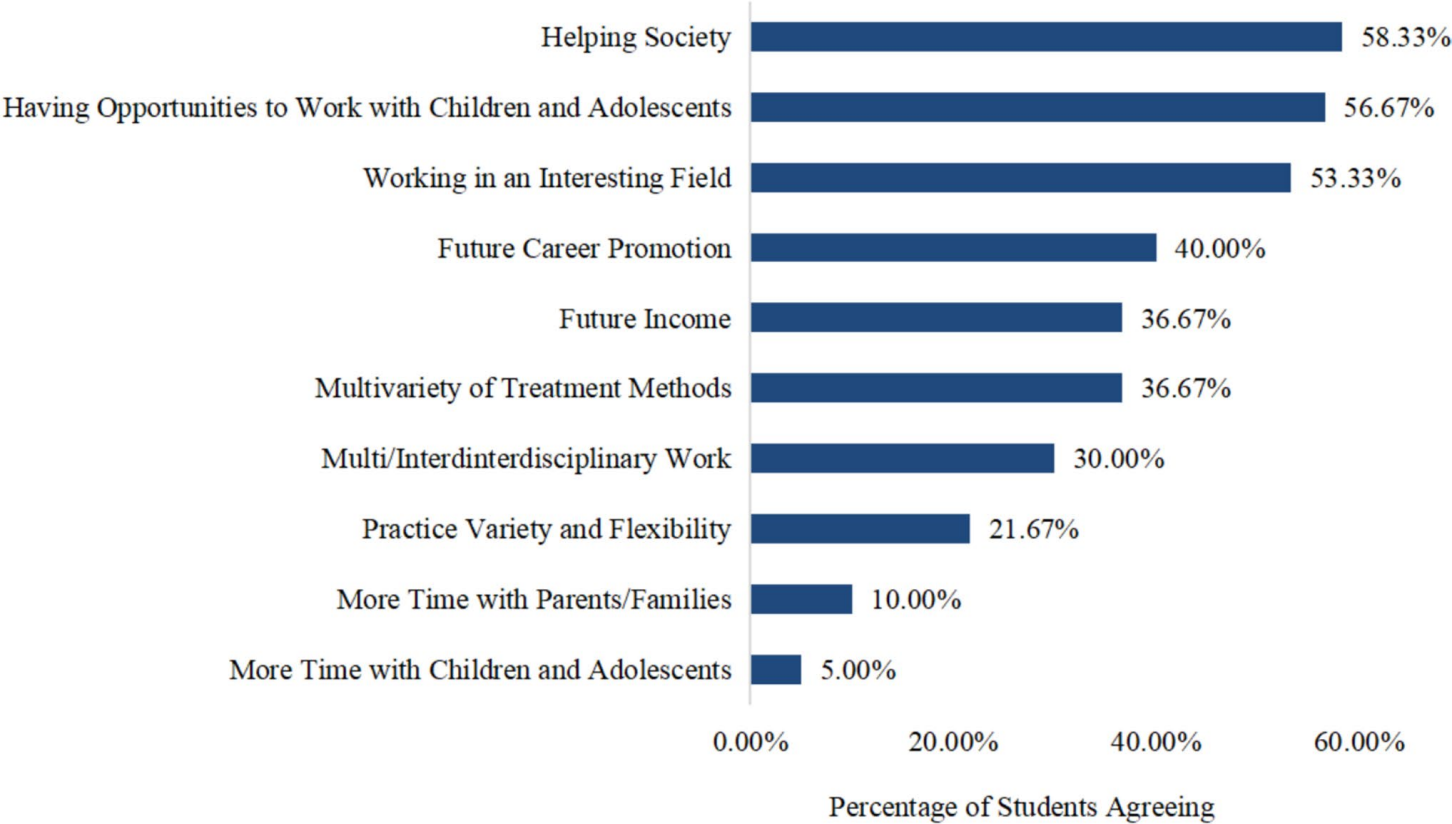
Attractiveness of CAP as a possible career choice (*N* = 60; multiple answers possible)

Among the 149 students, 100 (67.11%) had moderate to high interest in psychiatry, and 60 (40.27%) had moderate to high interest in CAP (≥ 6 points out of a maximum score of 10 points). To understand what influenced their interest in CAP, we asked this subgroup to choose attractive factors out of ten positive reasons. The top three reasons for pursuing a career in CAP included helping society, having the opportunity to work with children and adolescents, and working in an interesting field, with 58.33%, 56.67%, and 53.33%, respectively, of the students agreeing with the choices. The detailed aspects of CAP attractiveness and degree of career choice are listed in Fig. 2.

**Fig. 3:**
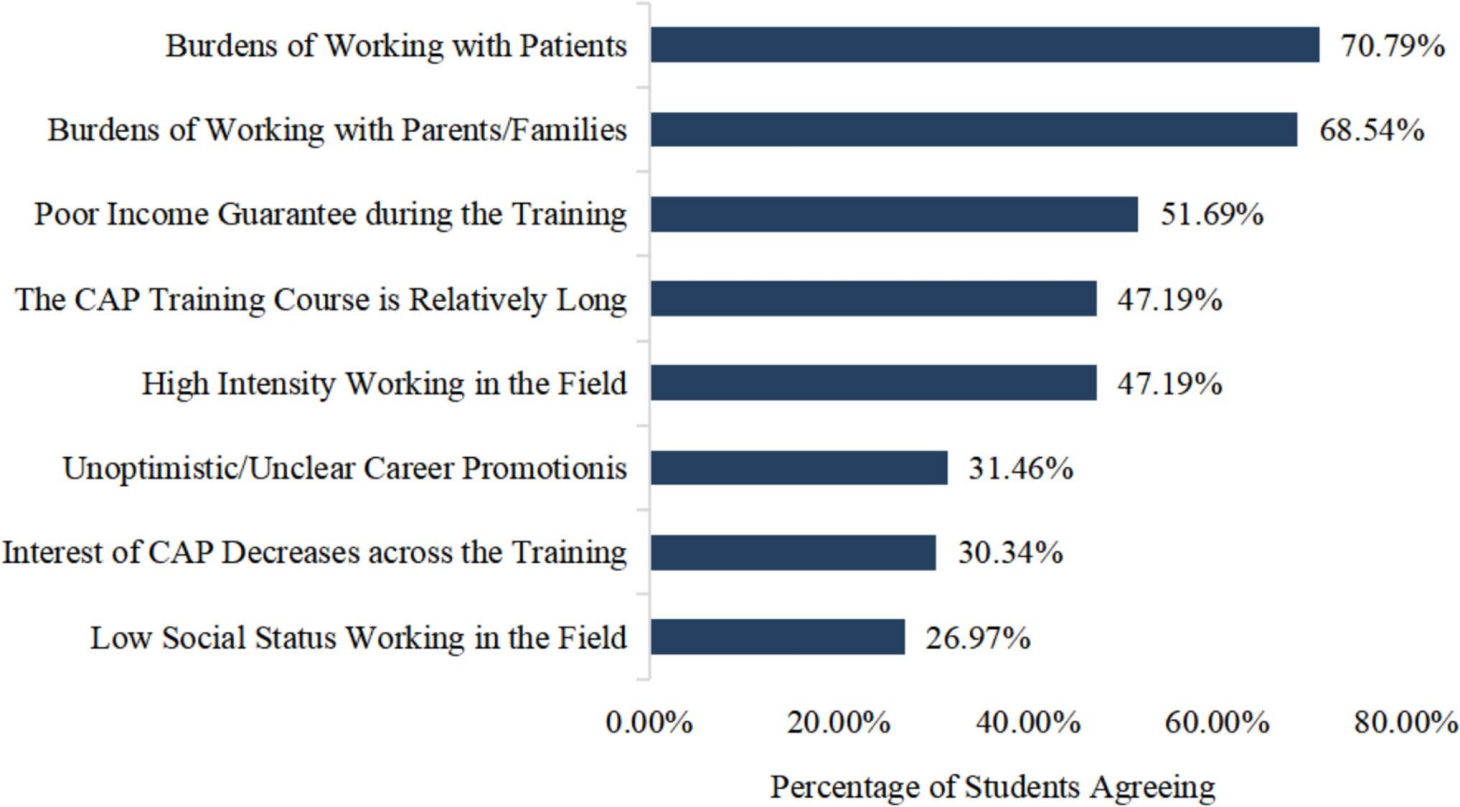
Obstacles to choosing CAP as a possible career (*N* = 89; multiple answers possible)

The other 89 students reported obstacles to CAP training, and the three leading factors were heavy burdens of working with children and adolescents, heavy burdens of working with parents, and poor income guarantees during CAP training, with 70.79%, 68.54%, and 51.69% of the students agreeing with the choices. The detailed aspects of obstacles to choosing CAP and their degree of career choice are listed in Fig. 3.

### Preference for CAP-Focused Curricula

**Fig. 4:**
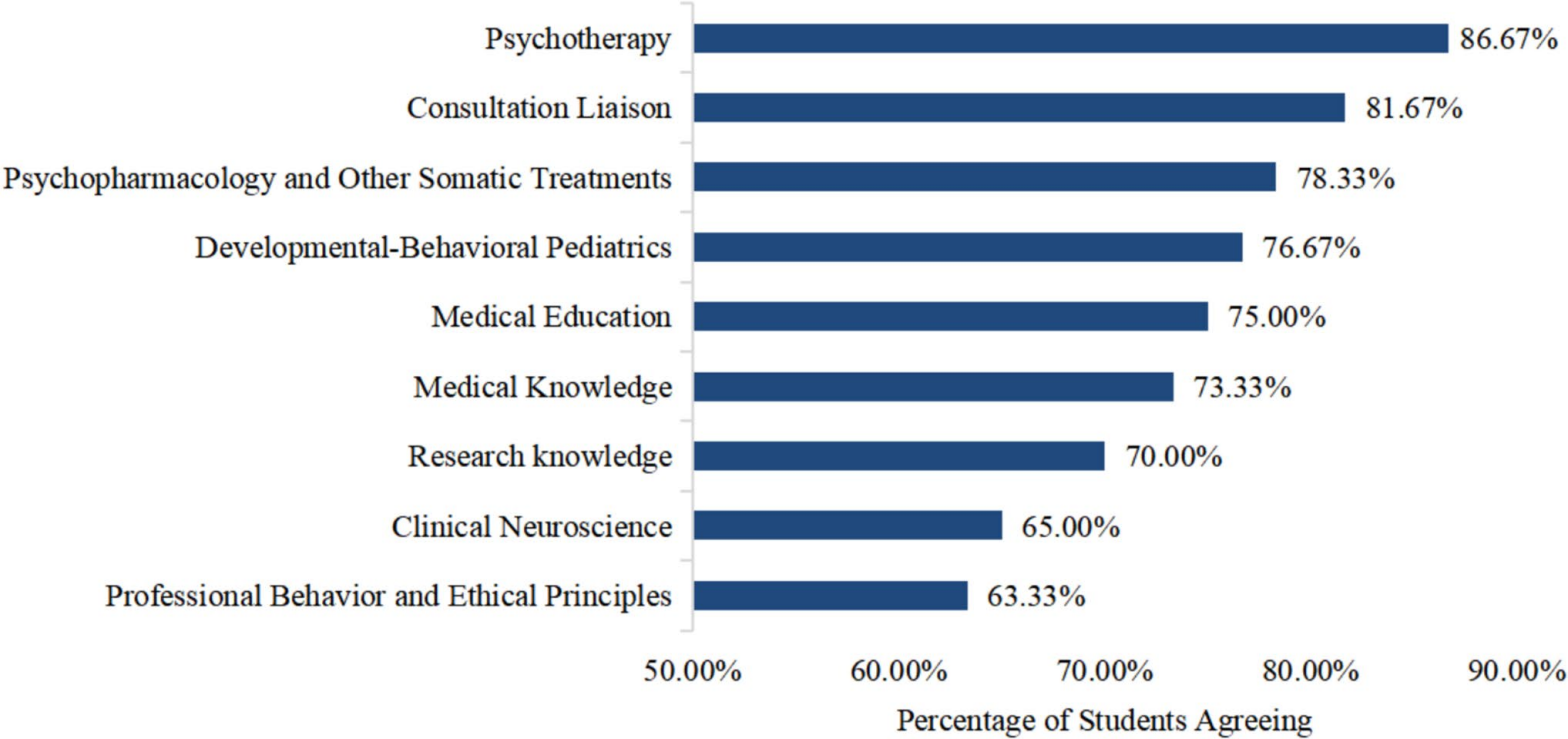
Attractiveness of CAP-focused curricula during undergraduate courses (*N* = 60; multiple answers possible)

Among the 60 students who demonstrated moderate to high interest in CAP, 48 (80.00%) expressed a desire to start learning relevant CAP knowledge during their undergraduate courses or even earlier, primarily through theoretical studies and clerkships (27 individuals chose, 45.00%) and clinical internships (19 individuals chose, 31.67%). In addition, curricula related to psychotherapy, consultation liaison, and psychopharmacology and other somatic treatments were identified as the most attractive areas, with high rating proportions of 86.67%, 81.67%, and 78.33%, respectively (see Fig. 4). In terms of preferred training formats, medical students were most likely to engage with CAP through clinical internships and practical training (51 individuals, 85.00%), followed by summer internships (34 individuals, 56.67%).

## Discussion

This is the first survey focusing on CAP career preferences among Chinese medical students. The survey achieved a high response rate of 88.17%, substantially higher than prior investigations [24, 26], thereby minimizing non-response bias. This study explored three dimensions: (1) current career choices and influencing factors, (2) attitudes toward choosing CAP as a specialty and influencing factors, and (3) preferences for undergraduate CAP curricula. The findings provide insight into what motivates medical students to pursue a career in CAP and suggest considerations for future curriculum development across undergraduate medical programs.

### Career Choice and Influencing Factors

Our study discovered that more than 80% of the students had already begun contemplating their future career directions, with 45.24% initiating this process during their second undergraduate year, even before their undergraduate education. Early exposure to various fields and interest-driven elective courses during the preclinical curriculum significantly impacts their future career decisions [27, 28]. This underscores the importance of multidimensional disciplinary introductions as early as possible in medical education to clarify career choices. In China, however, elective courses specifically focused on CAP are limited, indicating ample scope for further exploration and development in this area.

In addition, we found that career development and advancement, personal interests, and the fitness of personality and skills in the specialty were the top three aspects influencing the students’ career choices. This aligns with the previous results of a meta-analysis [29] and psychiatry career choice in Chinese medical students [23], highlighting that self-assessment of one’s preferences and specialty understanding are pivotal in shaping career decisions. Thus, curriculum design should integrate these influencing factors by providing inspiring early clinical exposure, refraining from undermining specific specialties, and transparently communicating career development and advancement policies to attract medical students to underserved specialties.

### Attractiveness and Obstacles to the Choice of CAP as a Possible Career

Our findings reveal that over half of medical students reported moderate to high interest in psychiatry, with nearly half showing similar interest in CAP. This contrasts with Wang et al.’s 2013 study, which presented a negative attitude toward psychiatry among responders [22]. The shift of attitudes may reflect growing societal and national emphasis on psychiatry, particularly CAP [30, 31], as we found only approximately one-fourth of the respondents acknowledged “the low social status of CAP” as a primary obstacle. This reflects increasing value and respect of psychiatry specialties in China.

The top three motivations for choosing CAP were (1) helping society, (2) having the opportunity to work with children and adolescents, and (3) working in an interesting field. The results were consistent with previous findings in psychiatry residents [26] and fellows [24]. Notably, helping society emerged as the top motivation. This motivation might reflect Confucian values of social responsibility, highlighting CAP’s cultural significance and societal impact in the Chinese context. Personal attitudes and interest toward CAP also played an important role in specialty choice. Given this information, early career initiatives—such as CAP learning groups, mentorship networks, and targeted promotions—could foster interest and provide exposure to CAP and enhance its appeal. Highlighting CAP’s societal benefits and rewarding aspects when working with children and adolescents may further strengthen its attractiveness.

The obstacles associated with the choice of CAP focus mainly on the burden of working with children and adolescents along with their family members. The possible reasons include managing complex symptoms, treatment implementation, high expectations, and considerations from family members [32], which contribute to burnout among child and adolescent psychiatrists [33]. To address these challenges, medical curriculum design should expand communication skills training for patients and family interactions, enhance students’ capacity to navigate complex clinical scenarios, and integrate diverse teaching methodologies such as scenario simulations and observational learning during clerkships or internships. Strategic promotional efforts can also highlight CAP’s appeal, thereby reducing the impact of related obstacles on students’ specialty choices.

### Preference for CAP-Focused Curricula

In our study, 80% of the students with moderate to high interest in CAP preferred learning relevant CAP knowledge during undergraduate courses or earlier, further emphasizing the importance of early exposure. Students preferred CAP-related learning through clinical clerkships and internships, providing ideas of practical formats for implementation. For example, medical schools may collaborate with affiliated teaching hospitals or other institutions to provide practical platforms, such as clinical and laboratory clerkships, internships, and other activities. These initiatives would help students gain clinical practice and scientific research, deepen their understanding of CAP, and foster interest in CAP-related careers.

Furthermore, students showed strong interest in interdisciplinary courses, particularly psychotherapy, consultation liaison, and psychopharmacology. This underscores the need to integrate psychiatry, pediatrics, psychology, and pharmacology into curriculum design. Such an interdisciplinary approach can help students develop a deeper understanding of CAP, identify synergies across disciplines, foster a developmental perspective on psychopathology, and facilitate the transition from theoretical foundations to clinical applications. Additionally, interdisciplinary practical programs complementing this curriculum can further enhance students’ ability to integrate and apply knowledge across fields, bridging the gap between theory and clinical practice.

The results of this study should be interpreted considering the following methodological constraints. First, our survey was limited to third-year medical students at Peking University Health Science Center, with a predominantly Han ethnic, male, high family income, and low economic burden demographic. These factors may restrict the generalizability of our findings and influence specialty choice. Future studies should conduct multicenter surveys across diverse regions, socioeconomic backgrounds, and academic years to enhance the generalizability of the findings and provide a more comprehensive understanding of trends in attitudes toward CAP throughout medical education. Second, the cross-sectional design limits the ability to establish causal relationships between early exposure to CAP and career preferences, and the results may not accurately reflect the trends in the preferences over time. Longitudinal studies tracking the same cohort over time in future studies would be valuable to estimate changes in attitudes toward CAP and identify critical points for intervention in medical education.

In addition, several potential biases and measurement considerations warrant acknowledgment. Despite the high response rate, students with a pre-existing interest in psychiatry or CAP may have been more likely to participate. The respondents might overreport their interest in CAP to align with perceived desirable or societal expectations. To address these, future studies could incorporate a comparative analysis of medical students’ interest in and perceptions of various specialties. This approach would help identify common factors influencing career choices across specialties and elucidate unique barriers and facilitators specific to CAP. Additionally, while personal interests and compatibility with the specialty emerged as key career determinants, current evaluations lack validated assessments of CAP-relevant individual characteristics. Future research should develop a multidimensional assessment system based on CAP core competencies for medical students [34, 35] to help them gain deeper self-recognition and, consequently, choose a more suitable profession. This would also streamline the process of recruiting highly suitable candidates for CAP training among medical students.

In conclusion, this study is the first to explore the medical students’ current career preferences in China, their attitudes toward choosing CAP as a specialty and related influencing factors, and their preferences for CAP-focused courses in medical education. These findings further highlight the significance of early exposure to CAP and provide valuable considerations for the future development of undergraduate curriculum systems, such as clinical clerkships, specialized internships, and interdisciplinary education. However, as a pilot study conducted at a single institution, these results require further validation through larger, multi-institutional research to establish the generalizability of the findings and the validity of the measures used. Despite these limitations, this study serves as an important foundation for indicating potential curricular changes at all stages of medical training that may encourage students to pursue careers in CAP. Educational institutions should consider optimizing course offerings, intensifying practical teaching, and fostering interdisciplinary integration to enhance medical students’ understanding of and interest in CAP. These efforts could better align with the career development needs of medical students and promote workforce development in the CAP field.

## Data Availability

The data that support the findings of this study are available on request from the corresponding author upon reasonable request.
